# Three-dimensional reduction method with a modified C2 isthmus screw in irreducible atlantoaxial dislocation: a technical note

**DOI:** 10.1186/s12893-021-01321-0

**Published:** 2021-08-12

**Authors:** Shengyuan Zhou, Bo Yuan, Weicong Liu, Yifan Tang, Xiongsheng Chen, Lianshun Jia

**Affiliations:** 1 Spine Center, Department of Orthopedics, Shanghai Changzheng Hospital, Naval Medical University (Second Military Medical University), Shanghai, 200003 People’s Republic of China; 2 Department of Orthopedics, the Second Affiliated Hospital, Hunan Normal University, Changsha, People’s Republic of China

**Keywords:** Atlantoaxial dislocation, Screw, Reduction, Fixation

## Abstract

**Background:**

Three-dimensional reduction plays a vital role in surgical reduction of irreversible atlantoaxial dislocation (IAAD). However, the most commonly used combination of C1 pedicle screw (PS) or lateral mass screw (LMS) and C2 PS or isthmus screw often fails to achieve satisfactory reduction at one time. The difficulty is usually caused by short anteroposterior and vertical distance between heads of C1 and C2 screws, which lack enough space for reduction operation. The objective of this study is to describe a three-dimensional reduction method with a modified C2 isthmus screw and to illustrate its advantage and effectiveness for IAAD.

**Methods:**

Twelve patients with IAAD underwent reduction and fixation with modified C2 isthmus screw combined with C1 PS or LMS, fusion with autologous bone graft. The insertion point was lateral to the intersection of caudal edge of C2 lamina and lateral mass, with a trajectory towards C2 isthmus, via lateral mass. The three-dimensional reduction was achieved through pulling and distracting. Radiographic evaluation included anteroposterior and direct distance between different insertion points, the occipitoaxial angle (O-C2A), clivus-canal angle (CCA) and cervicomedullary angle (CMA). Clinical outcomes evaluation included the Japanese Orthopaedic Association (JOA) score, Visual analog scale (VAS) and Neck Disability Index (NDI).

**Results:**

All the patients maintained effective reduction during the follow-up. The anteroposterior and direct distance was significantly higher in modified C2 isthmus screw than C2 PS whether combined with C1 PS or LMS (P < 0.05). The degree of O-C2A, CCA and CMA, JOA score, NDI, and VAS were significantly improved after the surgery (P < 0.05).

**Conclusions:**

Three-dimensional reduction method with a modified C2 isthmus screw is effective and safe in managing IAAD. It can increase the anteroposterior and vertical distance between the heads of C1 and C2 screws, which is benefit for the three-dimensional reduction operation of IAAD.

## Introduction

Cervical spinal cord compression caused by atlanto-axial dislocation (AAD) often leads to paralysis, even threaten the life of patients. The anatomical reduction is the key to relieve spinal cord compression. Reasons for AAD mainly include transverse ligament rupture, old odontoid fracture, Os odontoideum, rheumatoid arthritis, and atlantoaxial developmental deformities. AAD could be manifested as simple or complex dislocation in sagittal, coronal or transverse plane [1–4]. The dislocation in sagittal plane could be anteroposterior, vertical and angulated. For coronal and transverse plane, the dislocation is usually shown as atlantoaxial joint space asymmetry and rotatory dislocation respectively [5]. Spinal canal stenosis caused by anteroposterior AAD and compression on the cervical spinal cord or medulla oblongata caused by vertical dislocation of odontoid are the main causes of spinal cord compression. Therefore, the reduction of atlantoaxial joint in anteroposterior and vertical direction is the key of decompression.

In terms of the severity and difficulty in the reduction of the atlantoaxial joint, the dislocation is usually divided into reducible, irreducible and fixed AAD [6, 7]. For reducible dislocation, stabilization can be achieved by the screw-rod system, screw-hook system or cable tension band after reduction. For irreducible dislocation, the reduction of the atlantoaxial joint in three planes should be taken into consideration. The reduction would be failed if only anteroposterior AAD was paid attention without the correction of vertical and angulated dislocation. Vertical and angulated reduction are the same keys to success. So, the three-dimensional reduction plays a vital role in surgical reduction. However, at present, the most commonly used combination of C1 pedicle screw (PS) or lateral mass screw (LMS) and C2 PS or isthmus screw in the posterior approach often fails to achieve satisfactory reduction at one time. Multiple-adjustment of the depth between C1 and C2 screws [8], or rotation of rods, or lever mechanism is needed for reduction [9, 10]. The main surgical methods for irreversible atlantoaxial dislocation (IAAD) include anterior release and reduction with posterior fusion [11, 12], posterior occipitoaxial fusion [13], transoral atlantoaxial anterior decompression and fusion [15–17], etc.

**Fig. 1:**
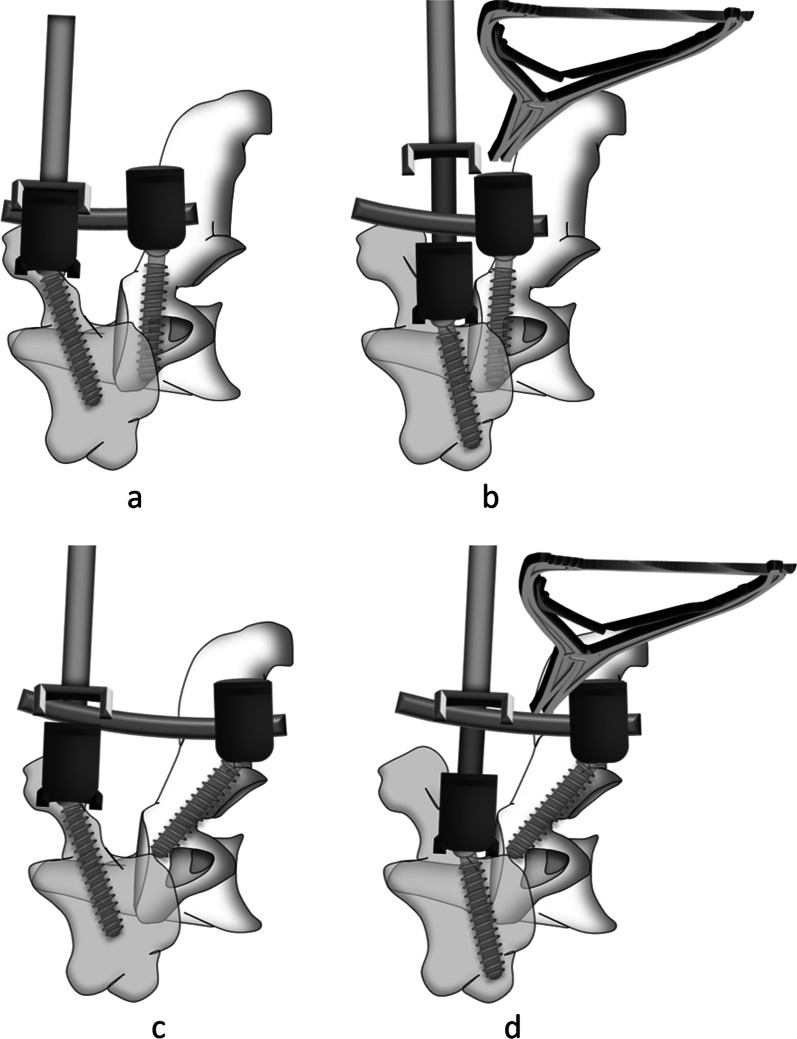
**a** C1 PS and C2 PS, with anteroposterior distance is too short to draw the C1 posteriorly. **b** C1 LMS and C2 PS, with vertical distance is too short to manipulate reduction for the vertical distraction. **C**–**d** C1 PS or LMS combined with modified C2 isthmus screw, with anteroposterior and vertical distance are both increased, which is benefit for the reduction. *LMS* lateral mass screw; *PS* pedicle screw

**Fig. 2:**
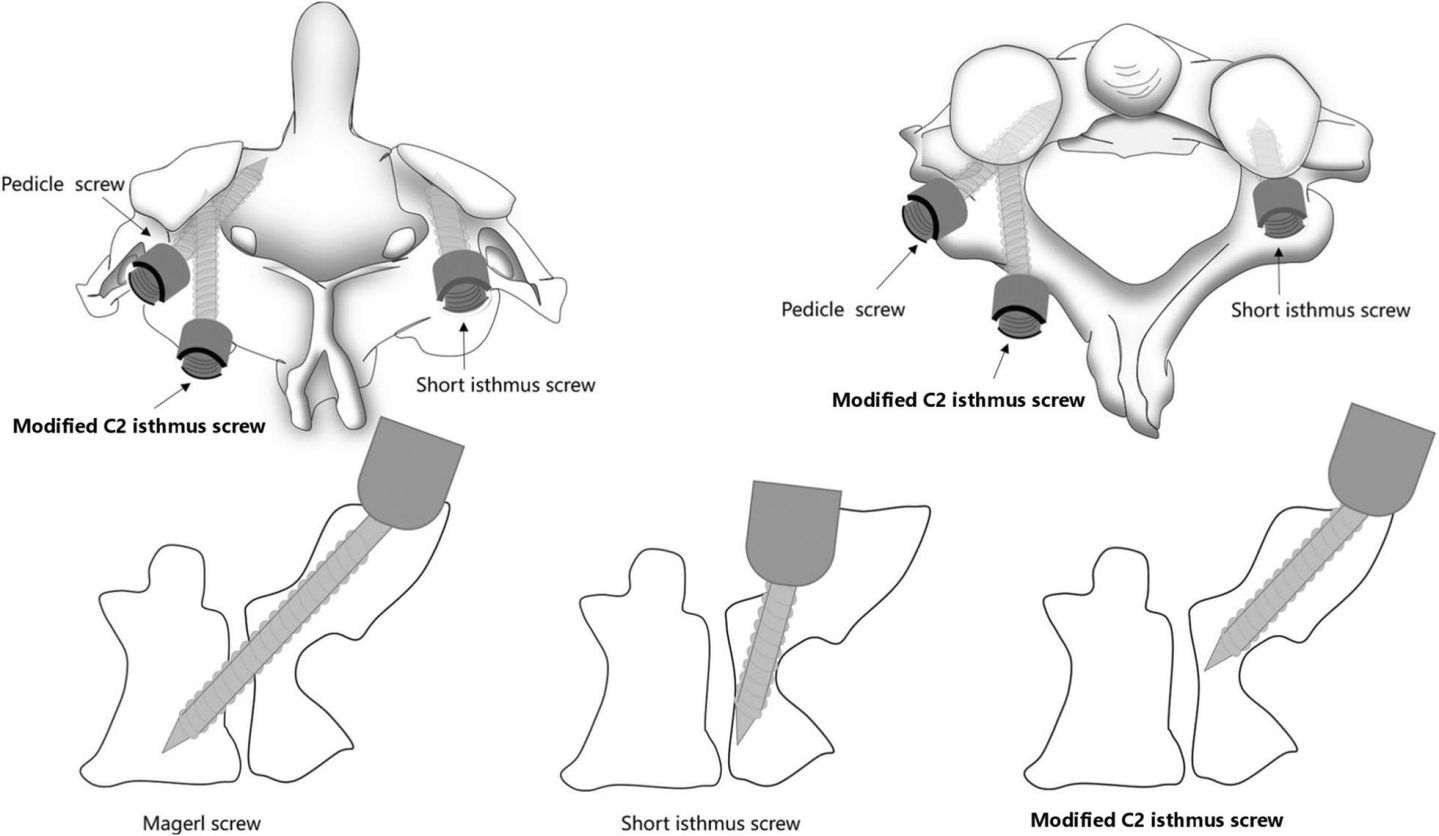
The difference between Magerl screw, short isthmus screw and modified C2 isthmus screw

The difficulty of reduction of IAAD was caused by the following two reasons: (1) the anteroposterior distance between heads of C1 and C2 screws is too short to draw the C1 posteriorly (Fig. 1a); (2) the vertical distance between heads of C1 and C2 screws is too short to manipulate the vertical distraction (Fig. 1b). Therefore, to increase the anteroposterior and vertical distance between the heads of C1 and C2 screws is the key to achieve atlantoaxial reduction. In our clinical practice, we modified C2 isthmus screw and set the insertion point at the intersection of caudal edge of C2 lamina and lateral mass, with a trajectory towards C2 isthmus, via lateral mass. The insertion point is to increase the anteroposterior and vertical distance between the heads of C1 and C2 screws, which is benefit for the reduction of anteroposterior and vertical dislocation (Fig. 1c, d). The modified C2 isthmus screw is different from the other techniques reported in the literature, such as Magerl screw [18] and short isthmus (par) screw [19, 20] (Fig. 2).

The objective of this study is to describe a three-dimensional reduction method with a modified C2 isthmus screw and to illustrate its advantage and effectiveness for IAAD. No studies to date have reported the details and outcomes on this technique.

## Methods

### Patient population

Twelve patients diagnosed as IAAD (6 males and 6 females) with a mean age of 43.8 years (range 13–66 years) were included in this study. All the patients underwent CT angiography to study the anomalous course of vertebral artery. The inclusion criteria included (1) patients with IAAD who cannot achieve complete reduction after skull traction; (2) cross-section of C2 isthmus was suitable for insertion of screw (width > 35 mm; height > 35 mm). Patients with vertebral artery variation (e.g. high riding vertebral artery) and severe osteoporosis, as well as patients unable to tolerate surgery, were excluded. The type of IAAD included Os odontoideum in 5, rheumatoid arthritis in 3, fracture nonunion in 2, fracture malunion in 1, and developmental malformation in 1. All procedures performed in studies involving human participants were in accordance with the 1964 Helsinki Declaration and its later amendments or comparable ethical standards. Informed consent was approved by the Institutional Review Board, and all subjects gave informed consent.

### Preoperative traction

**Fig. 3:**
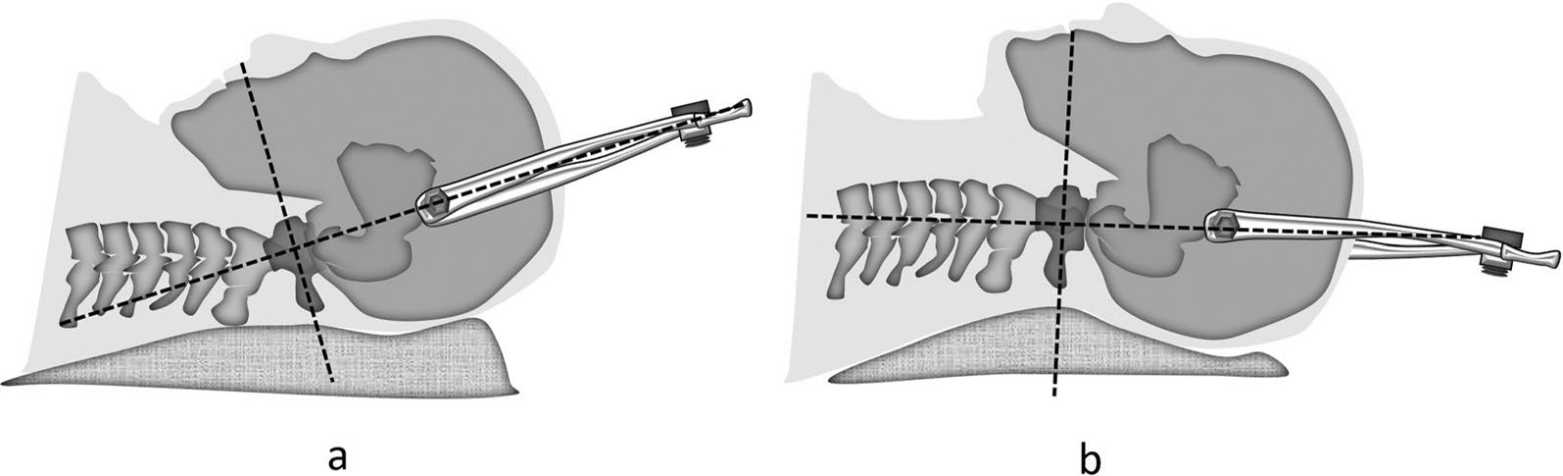
Traction direction is perpendicular to the anterior–posterior diameter of atlas. **a** Flexion position traction, **b** extension position traction

All the patients underwent skull traction under the electrocardiograph monitoring before the surgery. The skull traction was applied with a beginning of 3 kg and gradually adjusted to 10 kg (1 kg every 24 h) if the patients did not manifest as dyspnea or decline of muscle strength. The traction direction is perpendicular to the anterior–posterior diameter of atlas, and is adjusted from flexion (Fig. 3a) to extension (Fig. 3b) position gradually to loosen the structure which affecting reduction. The partial reduction was obtained in 11 cases while no reduction was achieved in one patient with malunion after fracture. The loosen of skull screw may occur and should be paid attention during the traction.

### Modified C2 isthmus screw

**Fig. 4:**
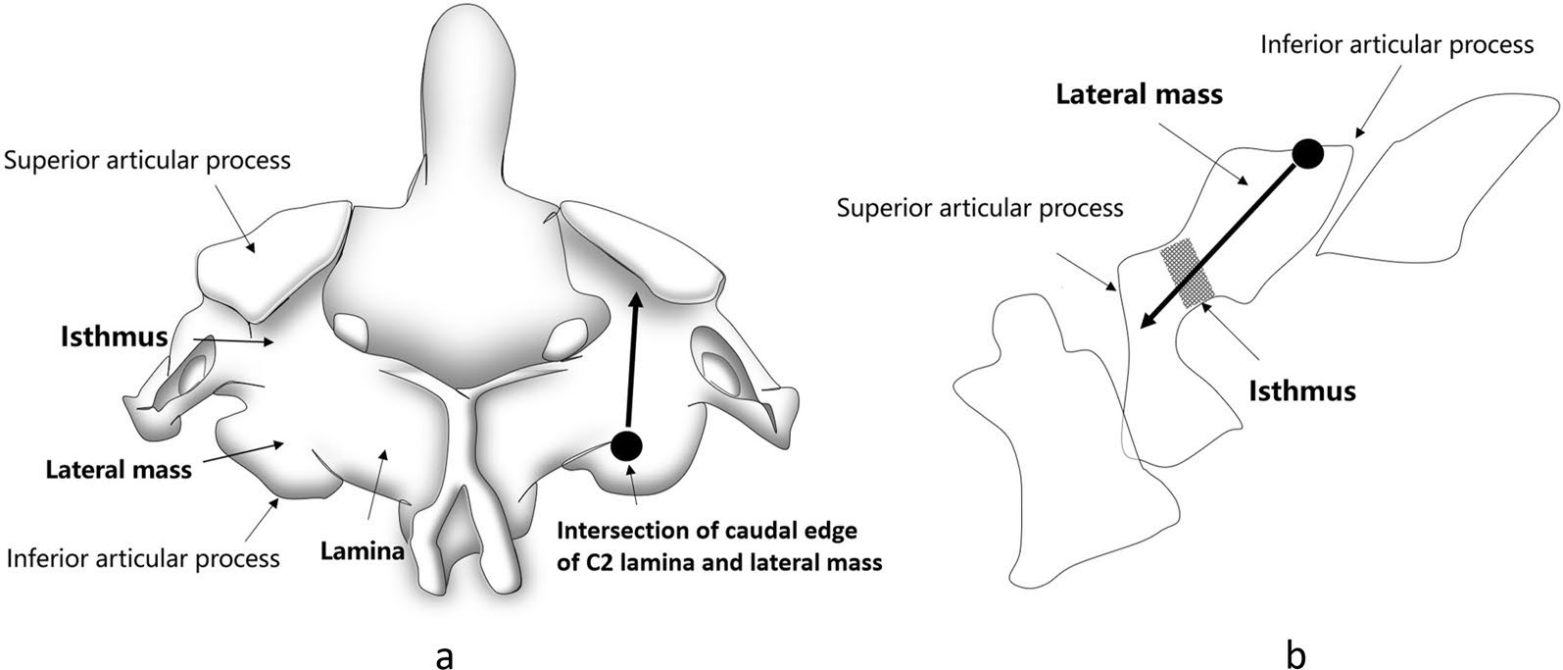
Insertion points of modified C2 isthmus screw. **a** Convergent direction, **b** cephalad direction

The entry point was the intersection of caudal edge of C2 lamina and lateral mass. The trajectory is in cephalad direction towards the C2 isthmus, via lateral mass (Fig. 4a). The anterior wall of C2 lamina and superior articular surface of C2 should not be injured or perforated (Fig. 4b).

### Reduction technique of AAD

**Fig. 5:**
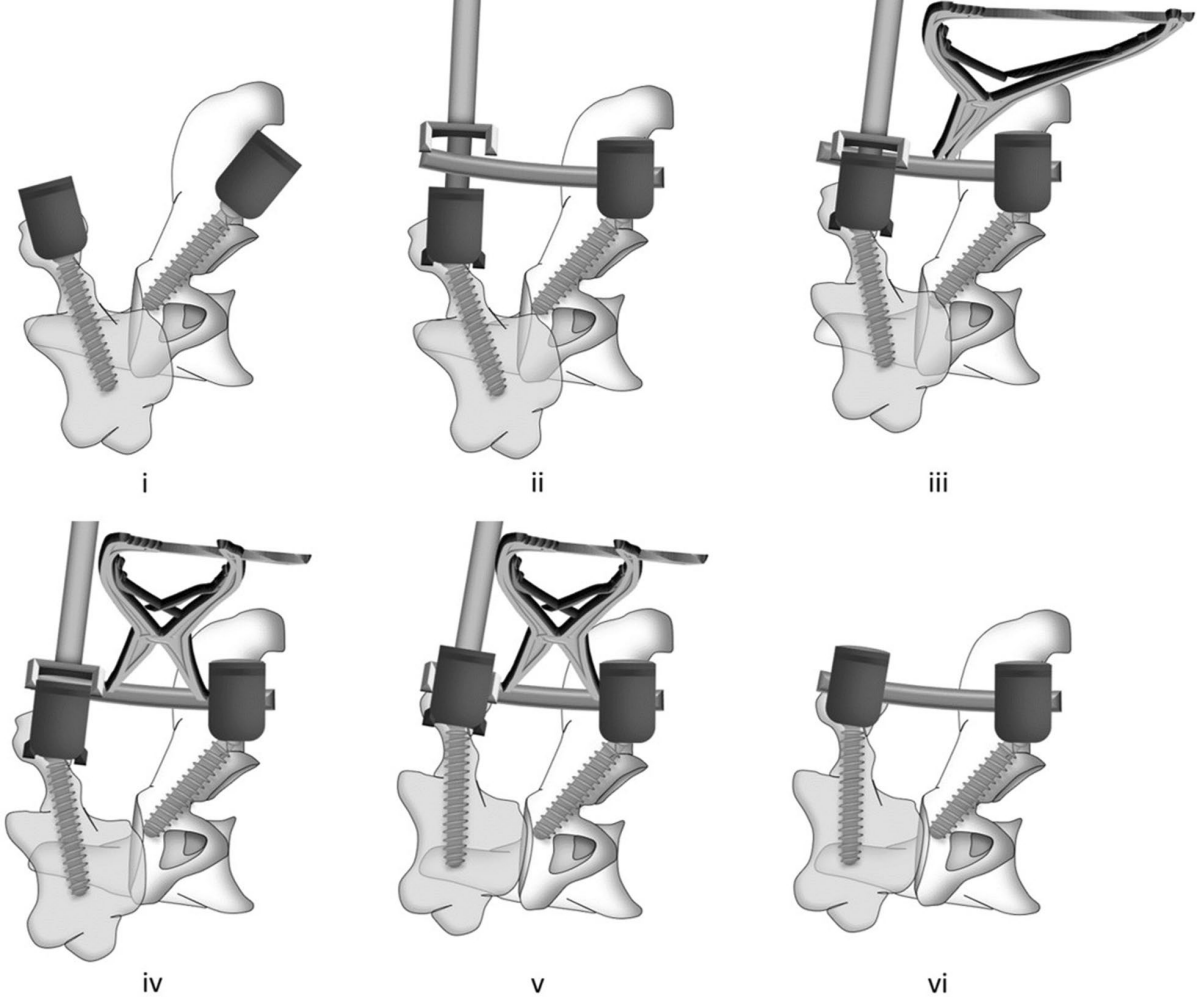
Reduction process: **i** Insert of C1 PS or LMS and modified C2 isthmus screw; **ii** fasten the pre-bent rod to modified C2 isthmus screw; **iii** pull the C1 and correct angulated dislocation; **iv** vertically distract; **v** achieve the reduction of lateral, rotational and vertical dislocation; **vi** fix the rod over screw heads and fusion with bone graft. *LMS* lateral mass screw; *PS* pedicle screw

**Fig. 6:**
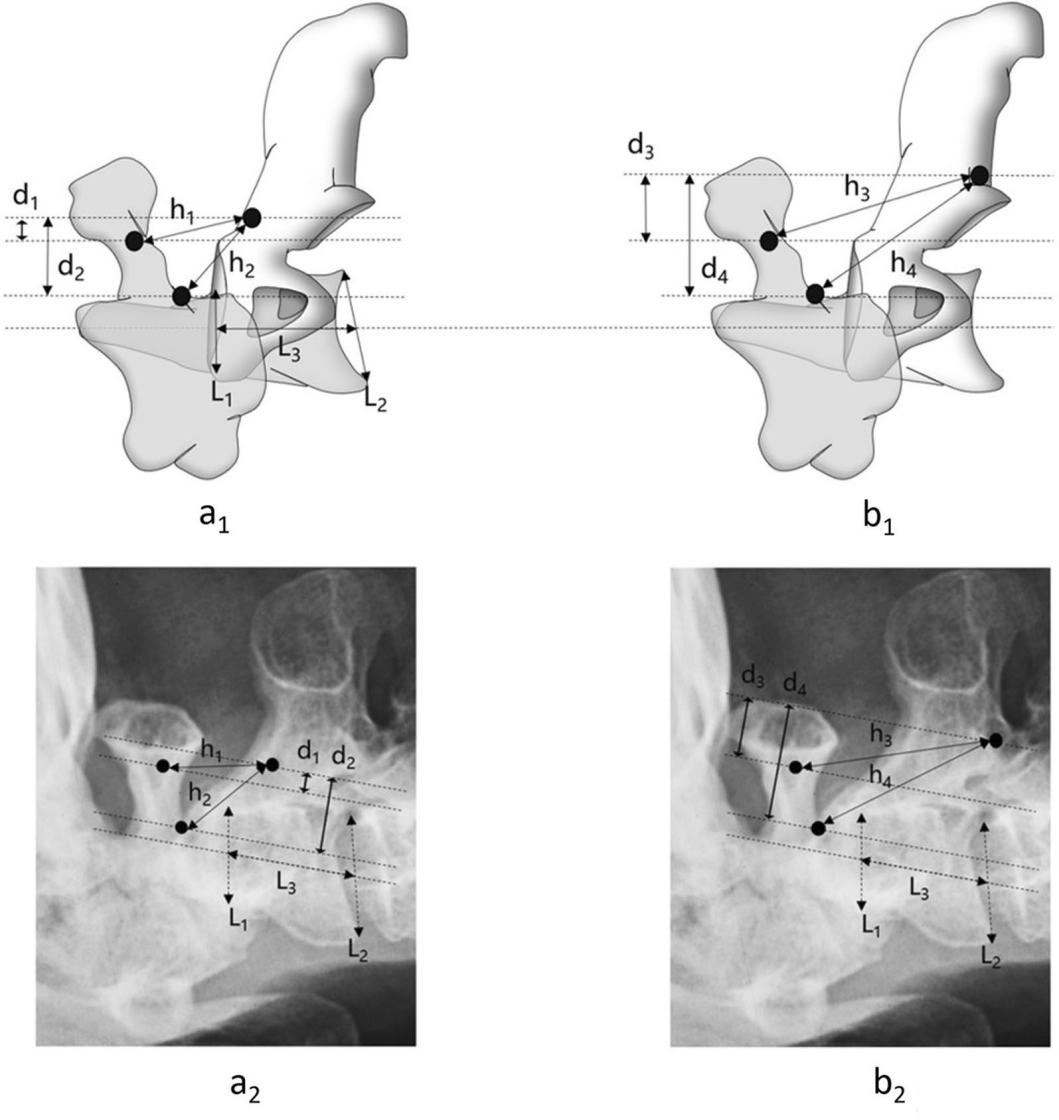
Measurement of the distance between insertion point of C1 and C2 screws. **a1** C1 PS or LMS combined with C2 PS on simulation diagram. **b1** C1 PS or LMS combined with modified C2 isthmus screw on simulation diagram. **a2** C1 PS or LMS combined with C2 PS on lateral X-ray. **b2** C1 PS or LMS combined with modified C2 isthmus screw on lateral X-ray. L1 indicates the base of odontoid process; L2 indicates inferior margin of C2; L3 indicates the line connecting the midpoint of L1 and L2. The measurement lines of HD are parallel to L3. APD and DD of C1 PS and C2 PS are d1 and h1; APD and DD of C1 LMS and C2 PS are d2 and h2; APD and DD of C1 PS and modified C2 isthmus screw are d3 and h3; APD and DD of C1 LMS and modified C2 isthmus screw are d4 and h4; with results showed d3 > d1, d4 > d2, h3 > h1, h4 > h2, which offer more operation space for reduction. *LMS*, lateral mass screw; *PS* pedicle screw; *APD* anteroposterior distance; *DD* direct distance

The reduction of anteroposterior, vertical and angulated dislocation on the sagittal plane is essential to the three-dimensional reduction of AAD. Reduction process: (1) Insert of C1 PS or LMS and modified C2 isthmus screw (Fig. 5i); (2) Fasten the pre-bent rod to modified C2 isthmus screw (Fig. 5ii); (3) Pull the C1 and partially correct anterior–posterior and angulated dislocation (Fig. 5iii); (4) Vertically distract and keep bilateral side of atlantoaxial joint symmetrical (Fig. 5iv); (5) Achieve the reduction of anteroposterior, vertical and angulated dislocation, and complete lateral and rotational dislocations simultaneously (Fig. 5v); (6) Fix the rod over screw heads (Fig. 5vi).

### Outcome evaluation

The anteroposterior and direct distance between heads of C1 and C2 screws have vital influence on pulling and distracting during the reduction. The anteroposterior and direct distance between the insertion point of C1 LMS or PS and C2 PS or modified C2 isthmus screw were measured on preoperative lateral X-ray by PACS system (AGFA, Mortsel, Belgium). On the lateral X-ray films, we set the insertion of C1 LMS at the junction of the posterior arch and lateral mass of C1; C1 PS at the junction of the posterior arch and posterior tubercle of C1; C2 PS at the junction of the isthmus and superior margin of lamina of C2; modified C2 isthmus screw at the junction of the inferior articular process and inferior margin of lamina of C2 (Fig. 6). The distance was used to reflect the operation space in the different combination of C1 and C2 screws during the reduction process.

**Fig. 7:**
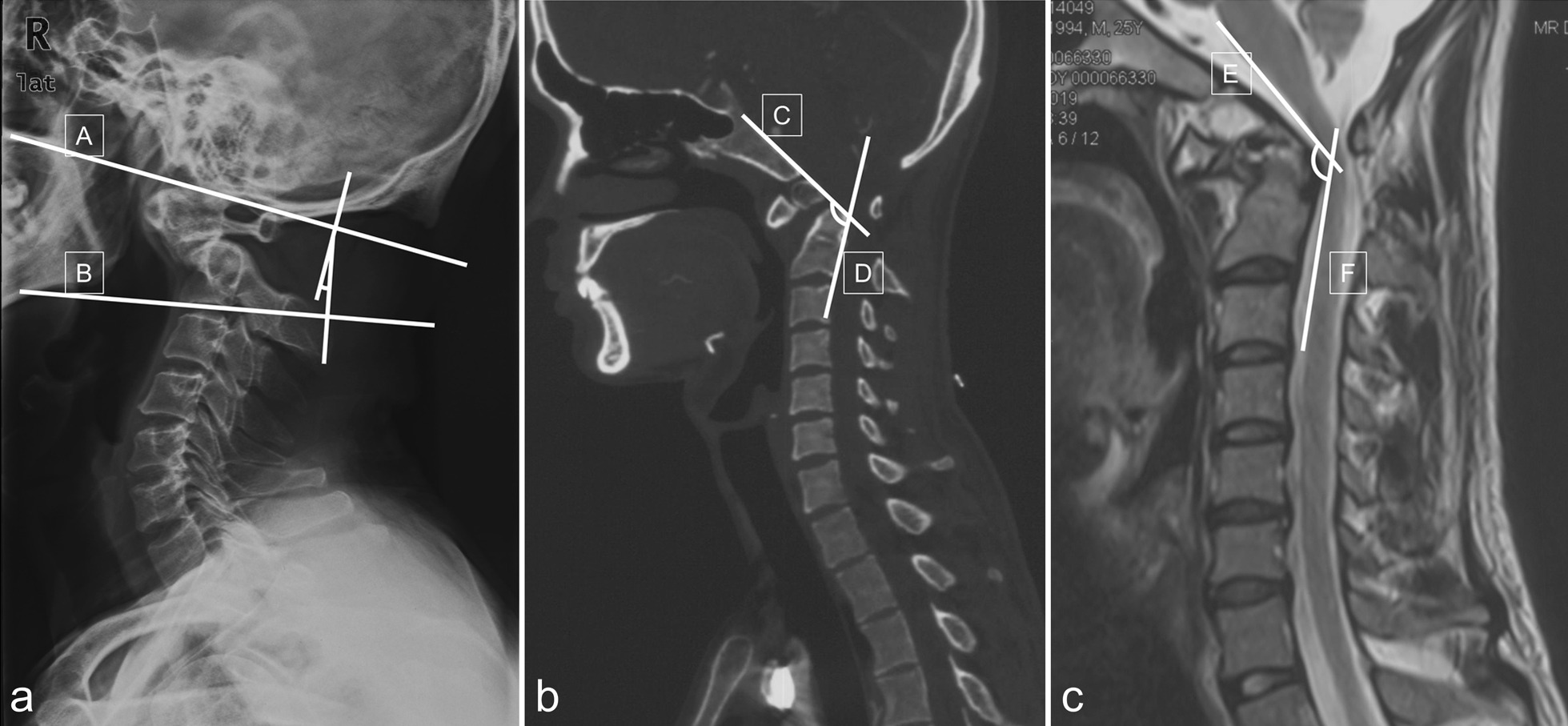
Measurement of the O-C2A, CCA and CMA. **a** O-C2A: Angle between McGregor line (A line) and the line (B line) tangential to the inferior aspect of the axis. **b** CCA: Angle of the line of inferior 1/3 clivus (line C) and the line extending from the posterior border of the dens to the posteriorinferior border of the axis body (line D). **c** CMA: Angle of the line extending the anterior border of the ventral medulla (line E) and the line extending the anterior border of the ventral upper cervical spinal cord (line F) in midline sagittal T2 MRI. O-C2A, Occipitoaxial angle; CCA, clivus-canal angle; CMA, cervicomedullary angle

The occipitoaxial angle (O-C2A), clivus-canal angle (CCA) and cervicomedullary angle (CMA) were measured to evaluate the reductive effect after surgery. The O-C2A defined by the angle between McGregor line and the line tangential to the inferior aspect of the axis [21] (Fig. 7A). CCA was defined as the angle between the line of inferior 1/3 clivus and the line extending from the posterior border of the dens to the posterior-inferior border of the axis body on the midline sagittal CT reconstruction images [22] (Fig. 7b). CMA was defined as the angle between the line extending the anterior border of the ventral medulla and the line extending the anterior border of the ventral upper cervical spinal cord on the midline sagittal T2 MRI [23] (Fig. 7C).

Clinical and radiographic data were obtained before and after surgery, and last follow-up. Clinical outcome assessment included the Japanese orthopedics association (JOA) score for neurological statue, Visual analog scale (VAS) for neck pain and neck disability index (NDI). Radiological examinations were performed before and after the surgery regularly to analyze the reduction and fusion. Bone fusion was defined as bridging bone across the bone graft interface on plain radiographs or CT scan.

### Statistically analysis

Paired t test was used to compare the difference. Statistical analysis was performed using SPSS version 16.0 (SPSS Inc., Chicago, IL). Significance was defined as P < 0.05.

## Results

### Surgical process

All the patients underwent reduction and fixation with C1 PS or LMS combined with modified C2 isthmus screw, fusion with autologous iliac bone graft. The C1–2 joints were not opened up in the surgery. For the patient with malunion after fracture nonunion, one-stage surgery including anterior osteotomy of odontoid through submandibular approach and posterior fixation C1 PS combined with modified C2 isthmus screw. The mean operation time was 132 min (range from 120 to 240 min), and the mean intraoperative bleeding loss was 168 ml (range from 100 to 600 ml).

### Radiological measurement

Preoperative measurement results of average anteroposterior and direct distance between the insertion point of C1 PS or LMS and C2 PS or modified C2 isthmus screw were shown in Table 1. The average distance was significantly higher in modified C2 isthmus screw group than C2 PS group whether combined with C1 PS or LMS (d1 5.6 mm vs. d3 12.5 mm and d2 13.8 mm vs. d4 20.7 mm in anteroposterior distance, h1 20.7 mm vs. h3 35.7 mm and h2 21.1 mm vs. h4 35.7 mm in direct distance, P < 0.05).

**Table 1:** General information and distance measurement of patients

Case	Age	Sex	Diagnosis	C1 PS + C2 PS		C1 LMS + C2 PS		C1 PS + modified C2 isthmus screw		C1 LMS + modified C2 isthmus screw		Follow-up duration, month
				APD, mm	DD, mm	APD, mm	DD, mm	APD, mm*	DD, mm*	APD, mm^#^	DD, mm^#^	
1	65	M	Fracture nonunion	3.8	20.8	14.6	21.5	11.5	36.9	22.3	37.7	86
2	66	F	Rheumatoid arthritis	3.6	21.4	10.4	19.3	10.7	37.1	17.9	35.0	82
3	24	M	Os odontoideum	7.0	24.5	14.5	22.5	14.5	37.0	21.5	35.0	32
4	29	M	Os odontoideum	7.4	25.8	14.2	22.6	18.9	36.3	20.0	34.2	85
5	63	F	Fracture nonunion	8.3	17.2	17.2	20.6	15.6	35.6	23.9	36.1	12
6	32	F	Os odontoideum	5.5	25.5	11.0	25.5	11.0	44.5	16.5	39.5	85
7	60	F	Rheumatoid arthritis	2.5	15.0	11.5	16.5	8.5	27.5	17.5	29.0	46
8	61	M	Rheumatoid arthritis	3.1	12.7	5.0	13.5	5.0	26.9	13.1	30.0	46
9	43	M	Fracture malunion	10.0	26.3	23.8	30.0	21.9	46.9	35.0	50.0	95
10	52	M	Os odontoideum	13.0	26.5	24.5	31.0	21.5	45.0	33.0	48.0	46
11	13	F	Developmental malformation	0.9	19.1	9.1	14.5	4.5	29.5	14.5	27.3	38
12	17	F	Os odontoideum	1.9	13.8	10.0	15.6	6.9	25.0	15.0	26.9	93
Average distance				5.6 ± 3.5	20.7 ± 4.5	13.8 ± 5.5	21.1 ± 5.5	12.5 ± 5.8	35.7 ± 7	20.7 ± 4.9	35.7 ± 7.1	62.2

*LMS* lateral mass screw; *PS* pedicle screw; *APD* anteroposterior distance; *DD* direct distance

*P < 0.001 compared with APD and DD in C1 PS + C2 PS respectively

^#^P < 0.001 compared with APD and DD in C1 LMS + C2 PS respectively

Radiographic measurement results before and after surgery were shown in Table 2. The mean degree of O-C2A, CCA and CMA before the surgery were 8.2°, 138.1°, 142°, respectively. The mean degree of O-C2A, CCA and CMA after the surgery were 18.1°, 148.7°, 155.2°, respectively, with significantly difference from pre-operative value (P < 0.05).

**Table 2:** Radiographic measurement before and after surgery

Case	Age	Sex	Diagnosis	OC2A		CCA		CMA	
				Pre-op, degree*	Post-op, degree*	Pre-op, degree^#^	Post-op, degree^#^	Pre-op, degree^&^	Post-op, degree^&^
1	65	M	Fracture nonunion	15.9	8.5	123.5	145.4	147.9	158.9
2	66	F	Rheumatoid arthritis	21.3	22.6	146.6	150	147	152
3	24	M	Os odontoideum	− 11.3	24.8	111.6	145.5	120.9	156.5
4	29	M	Os odontoideum	− 10.2	4.8	109.5	119.5	124.4	139.5
5	63	F	Fracture nonunion	− 4.6	11.1	143.3	158.2	146.1	153.1
6	32	F	Os odontoideum	− 3.8	16.6	126.5	136.5	143.9	154.2
7	60	F	Rheumatoid arthritis	17.3	20	152.3	156.2	147.4	150.2
8	61	M	Rheumatoid arthritis	28.4	26.9	155.9	171.3	152.3	166.7
9	43	M	Fracture malunion	1.8	14.6	145.9	150.4	144.8	163.3
10	52	M	Os odontoideum	10	18.3	151.3	158.5	143.6	150.2
11	13	F	Developmental malformation	9.9	16.9	144	151.2	129	164.7
12	17	F	Os odontoideum	24	31.5	146.5	142.1	156.8	152.6
Mean degree				8.2 ± 13.7	18.1 ± 7.7	138.1 ± 16	148.7 ± 12.9	142 ± 11.2	155.2 ± 7.5

*OC2A* O-C2 angle; *CCA* clivus-canal angle; *CMA* cervicomedullary angle

*P < 0.005 compared with pre-op OC2A

^#^P < 0.005 compared with pre-op CCA

^#^P < 0.005 compared with pre-op CMA

### Functional outcomes

The mean duration of follow-up was 62.2 months (range from 12 to 95 months). The atlantodental interval (ADI) is less than 3 mm on lateral X-ray and the compression on spinal cord have been released on MRI scan. All the patients showed clinical improvement in JOA (pre-op 10.4 ± 2.8, last follow-up 15.1 ± 1.3), VAS (pre-op 3.4 ± 1.6, last follow-up 1.5 ± 0.8) and NDI (pre-op 24% ± 12.3%, last follow-up 9.4% ± 4.3%)(P < 0.05) and returned to normal work. All patients fused with iliac bone autograft achieved fusion. Autologous bone was used in 2 cases and absorbed during the follow-up. But no hardware failure occurred at the final follow-up.

### Typical cases

**Fig. 8:**
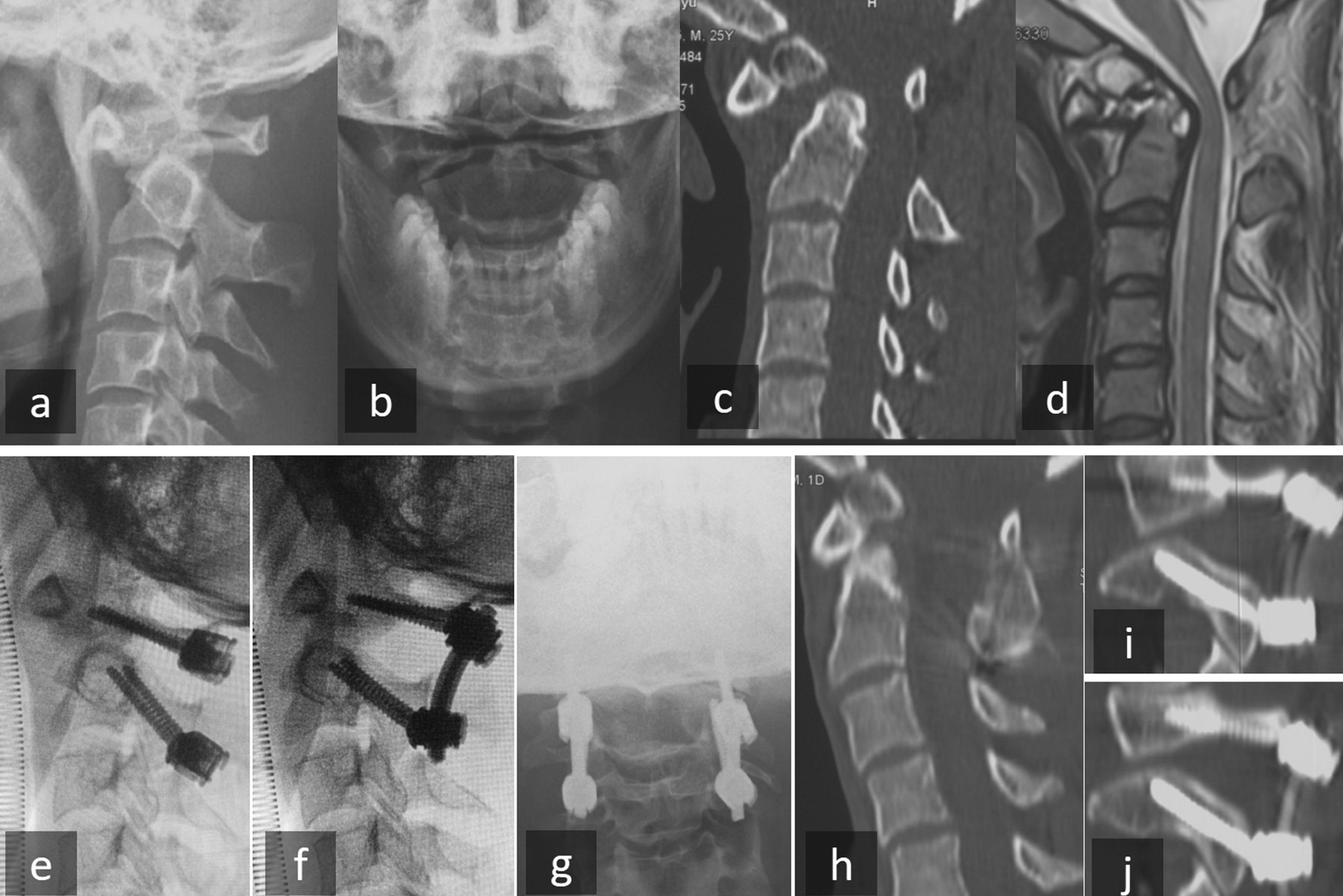
A 24-year-old man with Os odontoideum. **a**–**c** Preoperative X-ray and CT scan showed Os odontoideum and atlantoaxial dislocation. **d** Preoperative MRI showed spinal cord compression. **e** Intraoperative X-ray showed the position of C1 PS combined with modified C2 isthmus screw and **f** reduction. **g** Postoperative anterior–posterior X-ray, and **h**–**j** Postoperative CT scan showed good reduction and position of modified C2 isthmus screw. *PS* pedicle screw

**Fig. 9:**
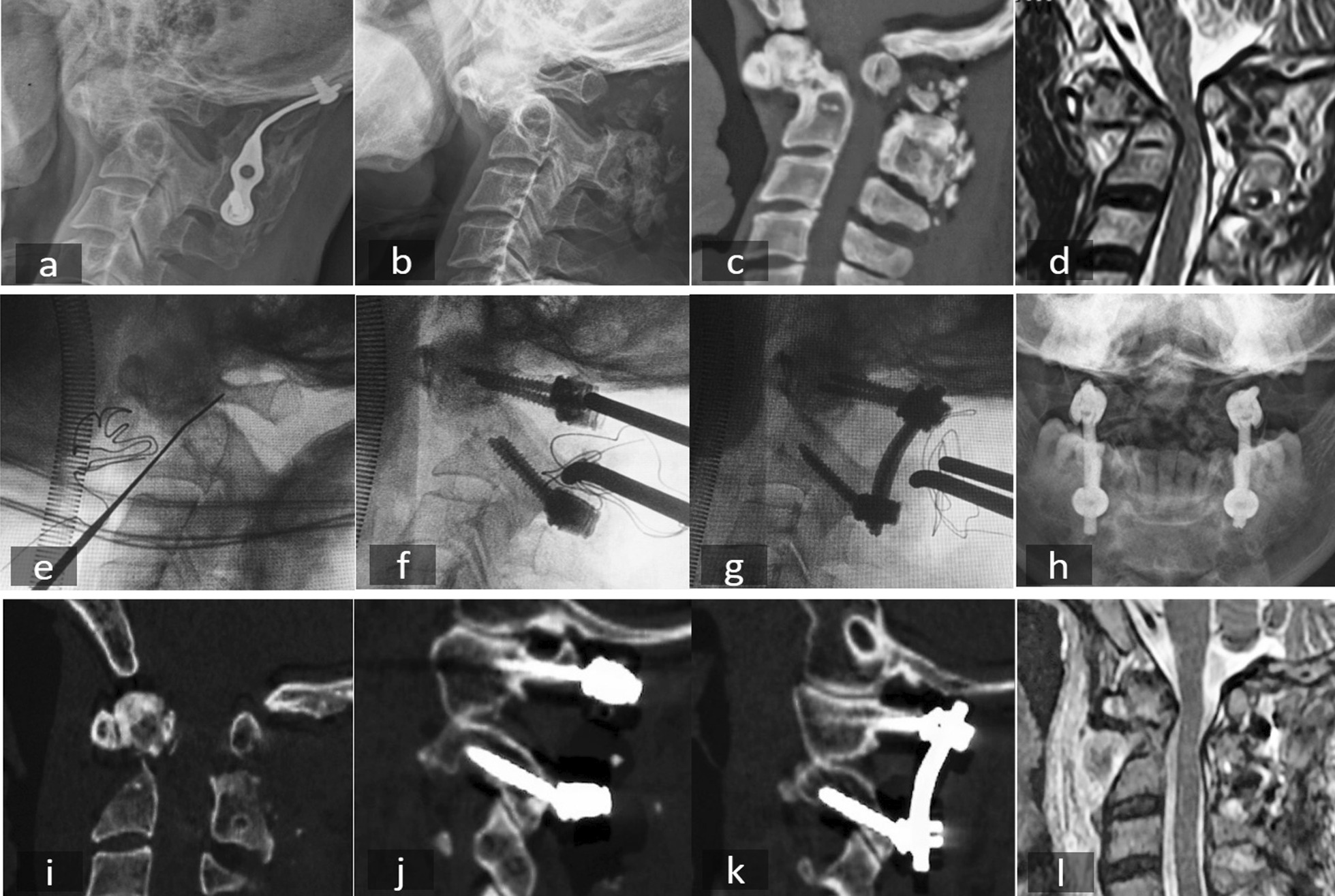
A 43-year-old man with fracture of C2 combined with atlantoaxial dislocation. **a** Preoperative X-ray showed C0-C2 bone graft and fusion was performed due to fracture malunion and dislocation of C1-C2 19 years ago. **b**, **c** Preoperative X-ray and CT scan showed malunion of C2 fracture. **d** Preoperative MRI showed atlantoaxial dislocation and spinal cord compression. **e** Intraoperative X-ray showed anterior release and osteotomy of odontoid. **f** Intraoperative X-ray showed the position of C1 PS combined with modified C2 isthmus screw land **g** Reduction. **h** Postoperative anterior–posterior X-ray, and **i**–**k** postoperative CT scan imagines showed good reduction and position of modified C2 isthmus screw. **l** Postoperative MRI showed the decompression of spinal cord. *PS* pedicle screw

Two typical cases were shown in Figs. 8 and 9.

## Discussion

The difficulty of the reduction of AAD is related to the atlantoaxial structure and the dislocation degree, as well as and the characteristics of the ventral tissue of atlantoaxial joint. For fixed AAD, it is essential for anterior odontoid osteotomy, release of atlantoaxial joint and removal of scars and osteophytes in atlanto-odontoid joint. The reducibility in most patients with congenital deformities is determined by the obliquity of joints. Salunke et al. described a technique of comprehensive drilling of C1–2 joints through a posterior approach to release them helps to achieve single stage reduction thereby circumventing the anterior procedure [24]. When there is no bony structure hinders the reduction, C1 PS or LMS combined with modified C2 isthmus screw could achieve reduction by vertically distracting between C1 and C2, and pulling C1 posteriorly. Anterior release of atlantoaxial joint is not necessary for this kind of patients. In our study, all the patients with IAAD got reduction after single-stage surgery by using C1 PS or LMS combined with modified C2 isthmus screw. The C1 posterior arch and the C2 lamina are fused with autologous iliac bone grafts after reduction. For patients without stenosis of vertebral canal caused by congenital anomalies of C1, there is no need to remove the C1 posterior arch for decompression. Odontoid osteotomy through submandibular approach, followed by posterior reduction and fixation with C1 PS or LMS and modified C2 isthmus screw was performed on the patient with malunion after fracture. No dysphagia or bucking was found after the surgery. No vertebral artery injury occurred in any patient. The O-C2A, CCA and CMA improvement showed that dislocations on anteroposterior and vertical planes achieved complete reduction, the neurological function improved s20nsion load than short isthmus screw. The intralaminar screw is usually used as remedial measures after the failure of other screws, rather than the first choice. Magerl screw provides sufficient resistance to flexion–extension load and is suitable for the fixation in atlantoaxial instability or dislocation after reduction, instead of reduction in IAAD [25]. Base on Magerl technique, modified C2 isthmus screw set the entry point at the intersection of caudal edge of C2 lamina and lateral mass, the trajectory is via lateral mass and towards the C2 isthmus, without perforating the facet joint of C1–2. The anteroposterior and vertical distance between the heads of C1 and C2 screws were increased, which is beneficial to the reduction of IADD. Up to now, there has no related biomechanical research about modified C2 isthmus screw, but its trajectory is similar with Magerl screw and short isthmus screw, which can resist flexion–extension load effectively. The trajectory of modified C2 isthmus screw is shorter than that of Magerl screw while longer than that of short isthmus screw. We speculate that its resistance to flexion–extension load falls in between the above two screws, which needs further confirmation. In our study, no hardware failure occurred at the final follow-up, which reflected that the modified C2 isthmus screw could provide sufficient biomechanical properties for the reduction and maintenance.

Same as C2 PS, the insertion of modified C2 isthmus screw is affected by the anatomical structure of C2 isthmus [26]. The normal C2 isthmus width and height is about 7–8 mm, which is enough for the placement of a 3.5 mm screw [27]. High riding vertebral artery is not rare in atlantoaxial dislocation patients [28, 29]. For this kind of patient, C2 transverse foramen often moves towards the medial and superior location, even occupies a large part of the isthmus. It is not enough for the screw when the isthmus is narrow. Trans isthmic screws are not suitable for such patients because the insertion may injure the vertebral artery. Therefore, the presence of narrow isthmus and a high riding vertebral artery is a contraindication for the C2 modified isthmus screw. However, the artery can be mobilized and a C2 subfacetal screw can be inserted for this issue [30]. Preoperative computerized tomography angiography for the vertebral artery and three-dimensional reconstruction of the cervical spine is indispensable. The evaluation of the relationship between the vertebral artery and C2 isthmus and measurement of C2 isthmus width and height is helpful to avoid vertebral artery injury.

## Conclusions

Three-dimensional reduction method with a modified C2 isthmus screw is effective and safe in managing IAAD. Modified C2 isthmus screw made some modifications on the basis of Magerl screw and short isthmus screw. It can increase the anteroposterior and vertical distance between the heads of C1 and C2 screws, which is benefit for the three-dimensional reduction operation of AAD shown as anteroposterior, vertical, and angulated dislocation in the sagittal plane, especially for irreducible cases.

## Data Availability

The datasets used during the current study available from the corresponding author on reasonable request.
